# Case Report of Transient Neonatal Hyperparathyroidism: Medically Free Mother

**DOI:** 10.7759/cureus.7000

**Published:** 2020-02-15

**Authors:** Eyad Almidani, Weam Elsidawi, Abdulaziz Almohamedi, Ibrahim Bin Ahmed, Abdulrahman Alfadhel

**Affiliations:** 1 Pediatrics, King Faisal Specialist Hospital and Research Center, Riyadh, SAU; 2 Pediatrics: Newborn Intensive Care Unit, King Faisal Specialist Hospital and Research Center, Riyadh, SAU; 3 Psychiatry, Alfaisal University, Riyadh, SAU; 4 Internal Medicine, Alfaisal University, Riyadh, SAU; 5 Emergency Medicine, Alfaisal University, Riyadh, SAU

**Keywords:** tnhp, transient, neonatal, hyperparathyroidism

## Abstract

Transit neonatal hyperparathyroidism (TNHP) is a very rare recessive mutation in the calcium channel transporter. TNHP is defined as an impairment of calcium transportation from the mother to the fetus prenatally and mainly in the third trimester. TNHP classically presents with skeletal deformities and subsequently affects multiple systems. TNHP has been linked to a mutation in the transient receptor potential cation channel, subfamily V, member 6 ​(​TRPV6). We report a case of a full-term male infant diagnosed with TNHP prenatally from a medically free mother. The patient was discharged home at the age of 28 days after an excellent response to the trial of calcium infusion.

## Introduction

Calcium is a mineral found naturally in food products. It is essential for many functions inside the human body. It is regulated by many factors such as the kidneys, parathyroid hormone, and gastrointestinal system. Unborn babies require many minerals. An essential mineral is calcium. Calcium is required for fetus skeletal formation and mineralization and is transported from the mother to the fetus through the placenta. It is transported through different mechanisms, one of which is transient receptor potential cation channel, subfamily V, member 6 (TRPV6). Mutations in this receptor have been identified, and it is a recessive trait that can cause transient hyperparathyroidism, which can subsequently cause skeletal deformities. Mutations in TRPV6 are rare phenomena, and only a few cases have been reported in the literature.

## Case presentation

We report a case of a three-week baby boy, the product of a full-term pregnancy and normal vaginal delivery. Apgar scores were seven, eight, and nine at one, five, and ten minutes after birth, respectively, His mother is medically free, Gravida 3 para 2+1. The birth weight was 3.2 kilograms. Previous pregnancies were significant for neonatal death at the age of 6 days; the baby had skeletal deformities, narrow chest, short, long bones, and lung hypoplasia associated with polyhydramnios. The working diagnosis was osteogenesis imperfecta (OI). A sample was sent to the OI panel, and the result was negative, with a first-trimester abortion in the second pregnancy.

In our case, the baby was born with an impression of OI, which turned out to be TNHP. Prenatally, the baby was found to have polyhydramnios with skeletal abnormalities; the impression was OI. The feto-maternal team did a trio whole-exome sequencing (WES)​ sample (index-chorionic villus sampling (CVS) and the parents), the index-CVS WES sample showed a homozygous variant of unknown significance in the TRPV6 gene (c.593C>G:p. T198R), which encodes for transient neonatal hyperparathyroidism. The father is heterozygous for the same variant, and the mother's status is unknown. After delivery, the baby cried immediately, and he was vigorous. Afterward, the baby was shifted to the newborn intensive care unit (NICU).

**Figure 1:**
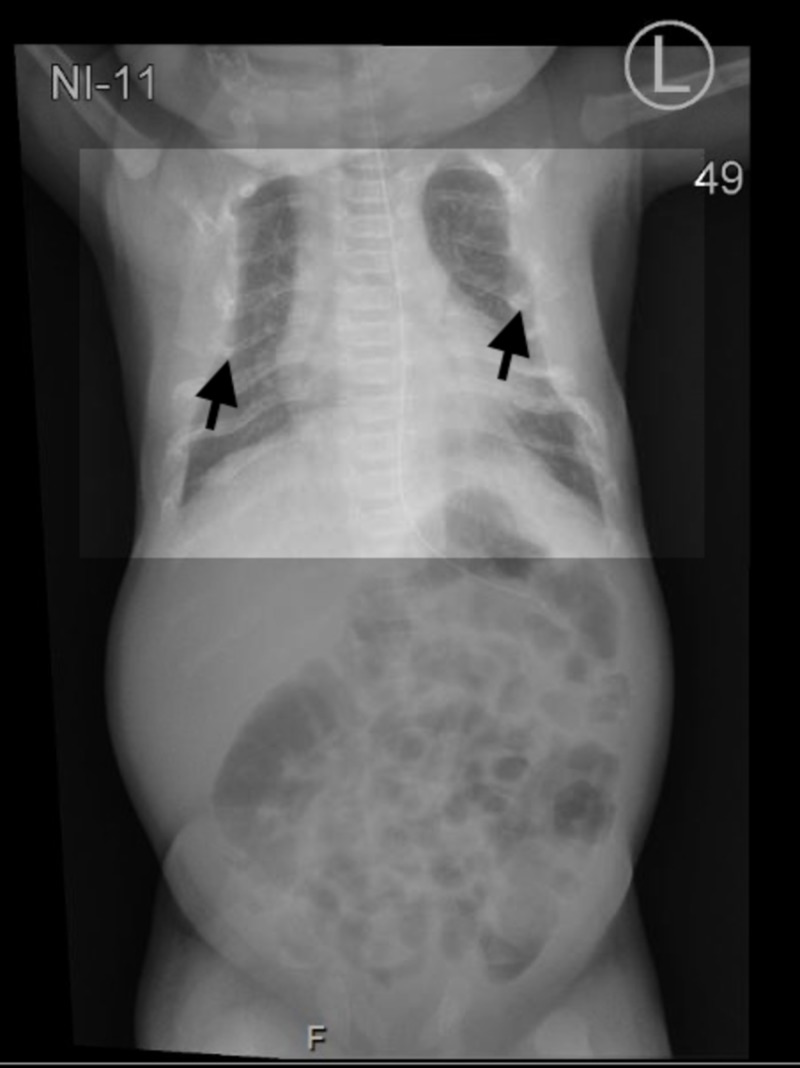
Chest X-ray showing a narrowed chest and multiple rib fractures

**Figure 2:**
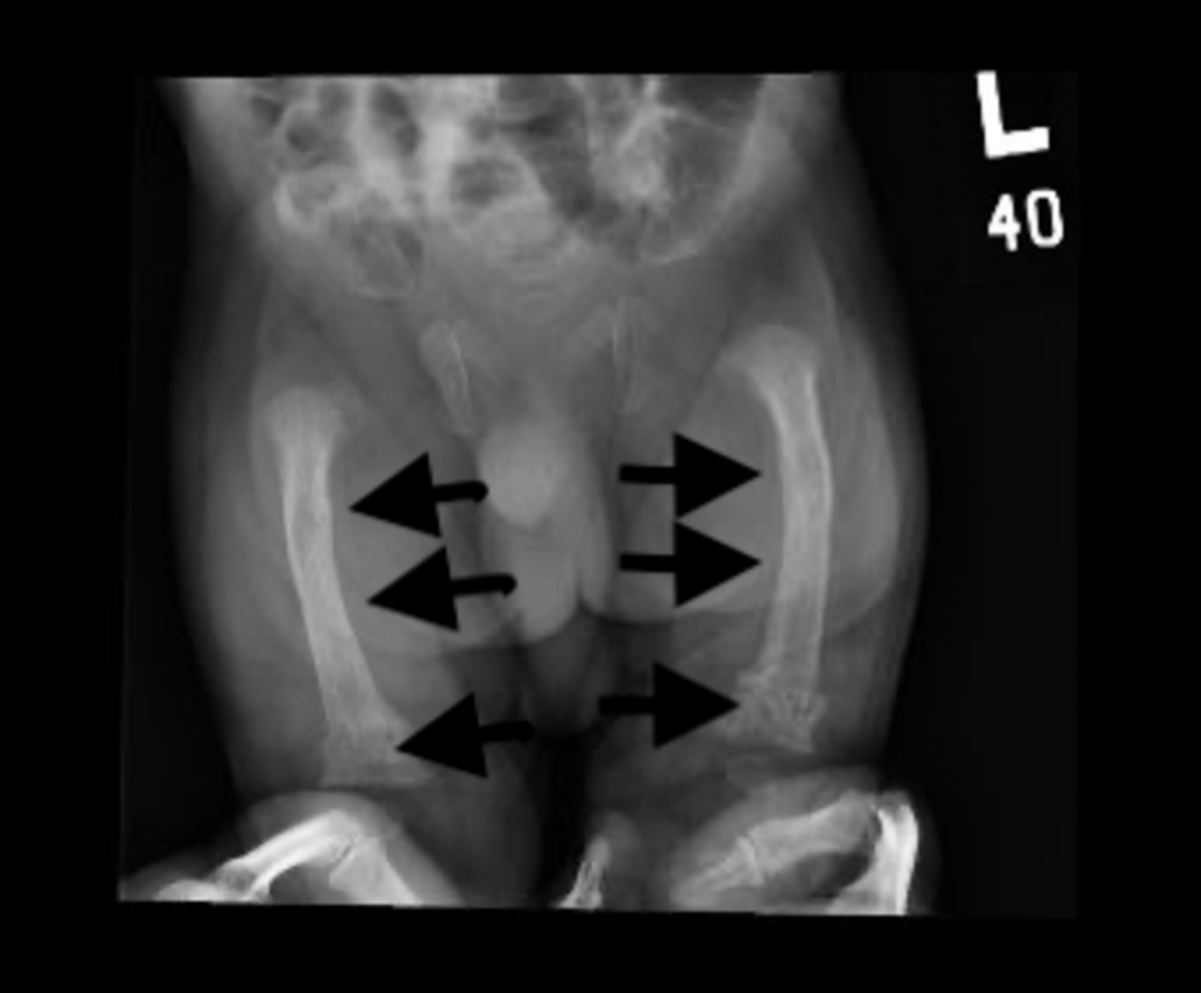
Hip X-ray showing bilateral bowing deformity of the femur and mildly impacted fracture of the distal femoral shaft bilaterally

Upon NICU admission, empirical antibiotics were initiated. Septic workup came up negative. A chest X-ray showed multiple rib fractures and a narrow chest (Figure 1). A hip X-ray showed generalized osteopenia associated with a bilateral bowing deformity of the femur and a mildly impacted fracture of the distal femoral shaft bilaterally (Figure 2). A hip spica was applied, and the pain management protocol was initiated due to the fractures. His oxygen saturation was 90% (normal oxygen saturation is 93%), which necessitates the use of the nasal cannula. Laboratory investigations revealed hypocalcemia (2.07 mg/dL), hypophosphatemia (1.25 mg/dL), and high alkaline phosphatase (249 IU/L). Initial parathyroid hormone (PTH) was significantly high at 813 pg/mL (normal range 10-56 pg/mL). A trial of calcium infusion daily was recommended by the pediatrics endocrinology team: elemental intravenous (IV) calcium infusion 1000 mg/m^2^/10 hours with close monitoring to avoid hypercalcemia, vitamin D 2000 IU, and phosphate 1.5 mmol BID.

**Figure 3:**
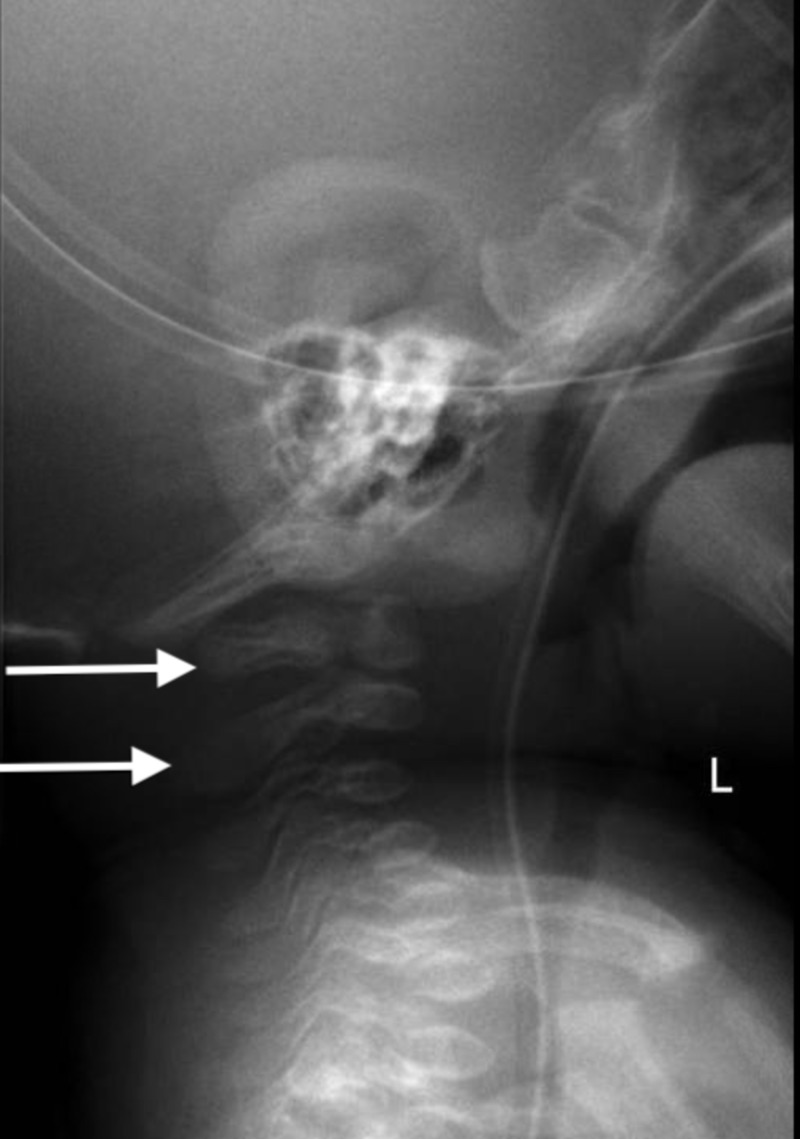
Pseudosubluxation of C2 and C3

A calcium infusion trial was initiated. Significant, gradual improvements in calcium, phosphate, alkaline phosphatase (ALP), and PTH were noted at the age of seven, 11, 14, and 19 days (Table 1). At the age of 14 days, skeletal survey results showed generalized osteopenia, pseudosubluxation of C2, and C3 (Figure 3). The PTH levels are now 68 with normalized calcium, phosphate, and ALP. It was recommended to stop the phosphate supplement because it was within the upper limit of the normal range.

**Table 1:** Response to the calcium infusion trial ALP: alkaline phosphatase; PTH: parathyroid hormone

Trial of calcium infusion				
Age	Calcium (N;2.1-2.6mg/dL)	Phosphate (N;1.4-2.3 mg/dL)	ALP (N; 122-469 IU/L)	PTH (N; 10-56 pg/mL)
7 days	2.1	1.86	270	450
11 days	2.4	1.8	315	364
14 days	2.5	1.5	400	53
19 days	2.7	2.03	430	68

At the age of 28 days, the baby boy was discharged home without any signs of respiratory distress. His hormonal panel was normal: calcium 2.47 ​mg/dL​, phosphate 1.45 ​mg/dL​, ALP 450 ​IU/L,​ and PTH 32 ​pg/mL​. A follow-up appointment was set after one month.

## Discussion

Our case is transient hyperparathyroidism caused by a mutation in the TRPV6 receptor. The mutation causes hypocalcemia in the unborn fetus, which in turn causes the body of the fetus to react to this phenomenon by breaking down the skeletal bones to retrieve calcium. The third trimester is the most important for bone growth. Abnormal bone findings can be found in the unborn fetus through ultrasound. When findings are noted in the ultrasound, differentials such as OI should be ruled out, as they have a similar clinical presentation. The only difference between the two diseases is the prognosis. For transient hyperparathyroidism, the baby can survive; however, OI carries, in general, a worse prognosis and later complications during life. OI is a rare inherited disease of connective tissue caused by mutations in some types of collagen. OI is mostly inherited as an autosomal dominant form, but some types are inherited in an autosomal recessive form. It is frequently associated with recurrent fractures and skeletal deformities but with no change in the parathyroid hormone levels [1-3].

TRVPV6 recessive mutations cause hypocalcemia by interfering with placenta-maternal-fetal calcium transport. Moreover, by this hypocalcemia, it causes secondary hyperparathyroidism and skeletal abnormalities. Because of impaired mineralization and increase bone resorption. According to the literature, only a few cases have been reported. They all presented with skeletal abnormalities and respiratory distress. Skeletal anomalies included generalized osteopenia, narrow chest, short ribs with multiple fractures, and bowing of long bones. Affected babies might require ventilator support for the first weeks to months of life, and they might require tube feeding. When a patient is suspected of having this mutation, a skeletal survey, genetic testing, and hormonal panel should be performed. Confirmatory genetic testing using WES can reveal the disorder, and appropriate management can be put in place [1-3].

After birth, sources of calcium changes from the placenta to the neonatal intestines. Calcium transport in the intestine, in general, by two mechanisms: transcellular and paracellular. Because of calcium absorption, in these cases, is predominantly maintained by the paracellular pathway, after giving adequate calcium, clinical symptoms of transient neonatal hyperparathyroidism should be resolved [2].

## Conclusions

TNHP management necessitates the early diagnosis, infusion of calcium, and close monitoring by a multidisciplinary approach. As presented in the case, TNHP patients respond very well to the trial of calcium infusion with a very good prognostic factor.
